# The use of BCAP in Finland: toward correct classification rate

**DOI:** 10.1080/02813432.2022.2139478

**Published:** 2022-11-03

**Authors:** Paavilainen Eija, Ellonen Noora, Mielityinen Laura

**Affiliations:** Faculty of Social Sciences, Tampere University, Tampere, Finland

**Keywords:** Child abuse risk, risk assessment, validation process of Brief Child Abuse Potential inventory, universal services, parental worries

## Abstract

**Objective:**

Further validation of Brief Child Abuse Potential (BCAP) inventory, for setting the correct classification rate.

**Methods:**

Data collection from potential abusers (*n* = 47), visiting in the hospital clinic meant for parents having special needs due to problems with alcohol and drugs connected to other evidence-based risk factors of child abuse. The risk level was compared between these 47 parents and previously collected data from 450 parents, representing general population.

**Results:**

There were no differences between likely abusers and the general population. Among both groups, 6% had elevated abuse risk and there were no differences in appearance of dimensions including in the abuse scale.

**Conclusions:**

Assuming child abuse based on known risk factors is not enough, when setting the correct classification rate. We need more accurate knowledge about the abuse, and the family life situation in general. However, assessing risk factors of child maltreatment systematically with the BCAP, can still serve as a fruitful basis of assessing parents’ needs and worries as the basis for providing support what they need.

## Introduction

In a previous article, we analyzed validity of the Brief Child Abuse Potential (BCAP) inventory among general population in Finland [1]. BCAP is one of the most frequently used measures in several countries and cultures for detecting potential child abuse [2]. The BCAP is a short version of original CAP inventory created by Joel Milner in 1986, which was validated in the Finnish context [3,4]. The BCAP is a self-report measure, of which an abuse risk scale can be calculated which constitutes of six sub-scales: Distress, Rigidity, Unhappiness, Problems with Child and Self, Problems with Family, and Problems with Others. In addition, the BCAP includes several validity scales to evaluate the reliability of the responses [5]. In different countries and cultures, the classification of child abuse rate differs, which needs to be taken account while calculating the risk level and assessing the family needs for services [2].

Our analysis concluded that the BCAP could be considered a reliable, quick and useful clinical tool for screening potential child maltreatment among parents [1,6]. However, our validation did not include an analysis of the correct classification rate, and did not utilize a comparison group of known abusers. To validate this type of tool and to get closer the correct classification rate, it is necessary to utilize data from known abusers, as a next step [2]. Therefore, these findings are presented in this paper. We first briefly present the main findings of the validation of the BCAP in a general population as a context for the current analysis.

### The BCAP among Finnish general population

On average, scores in abuse scales, indicating the risk of potential child abuse, were significantly lower in the Finnish sample compared to American samples or samples from other European countries [2,5]. Cut-off for abuse risk was therefore modified according to the manual of original CAP to be lower than in other studies. According to that cut-off, 6% of general population were classified as potential abusers [1].

Also, response validity to the scale varied in the Finnish sample compared to samples from other countries. Whereas 5.9% of responses were evaluated as invalid in Finnish sample based in validity index number of invalid responses varied between 27% and 31.9% in the American an UK samples [2]. Whereas the original CAP consisted of a six dimension structure (Distress, Rigidity, Unhappiness, Problems with Child and Self, Problems with Family, and Problems with Others), in the Finnish sample, a five-factor structure was the most suitable when eigenvalues, a contribution provided by the factors to the overall explained variance, and interpretability were considered. Those factors were: Loneliness and Distress, Impact of Others, Family Conflict, Rigidity and Financial Insecurity [1].

### Data

Data for estimating the correct classification rate (*n* = 47 parents) were collected in one hospital, at out-patient clinics providing specialized care to parents, including those who are expecting a baby, who have special needs due to problems with alcohol and drugs connected to other evidence-based risk factors of child abuse [1].

## Results

Among the assumed risk group (*n* = 47), 6.4% reported an elevated score on the abuse risk scale, which is equal to the rate among the general population sample. Of the respondents, 12.8% were evaluated as invalid based on the validity index, which was higher than in the general population sample (5.9%).

In Table 1, the means of the different dimensions included in the abuse scale are compared between likely abusers and the general population (*n* = 450). The rate of family conflicts and impact of others were slightly higher among likely abusers but none of the differences were statistically significant.

**Table 1.** Comparison of dimensions of an abuse scale between likely abusers and general population (*m*, SD).

	Likely abusers (*n* = 47)		General population (*n* = 450)		
	*m*	SD	*m*	SD	Range
Loneliness and distress	0.6	1.56	0.5	1.25	0–9
Impact of others	0.39	0.93	0.19	0.72	0–6
Family conflict	0.39	0.61	0.21	0.52	0–3
Rigidity	0.21	0.51	0.28	0.59	0–3
Financial insecurity	0.04	0.2	0.04	0.19	0–2

## Discussion

Evaluating the correct classification rate is important in validation of new tools for identifying potential child abusers. The correct classification rate of known abusers and non-abusers ranges from 80% to 90% in the original CAP [2,5]. In this paper, we evaluated the correct classification rate by analyzing the BCAP among likely abusers. The assumption that this group was likely abusers was based on them being patients in a special needs out-patient clinic, meant for parents having alcohol or drug problems and other known child abuse risks.

There were no differences between likely abusers and the general population. Among both groups, 6% had elevated abuse risk and there were no differences in appearance of dimensions including in the abuse scale.

In conclusion, it seems that assuming child abuse based on known risk factors is not enough, when setting the correct classification rate. We need more accurate knowledge about the abuse, and the family situation in general. However, assessing risk factors of child abuse systematically with the BCAP, can still serve as a fruitful basis of assessing parents’ needs as the basis for discussions with parents and for providing support, also based on worries which parents express by answering the BCAP instrument and what they bring into discussion while visiting in services [1,6,7].
